# Supplementary material for: DynaTag for efficient mapping of transcription factors in low-input samples and at single-cell resolution
Source: Nat Commun. 2025 Jul 28;16:6585. doi: 10.1038/s41467-025-61797-9 (PMC12304361; doi:10.1038/s41467-025-61797-9)
Supplement: Supplementary file 1 — Supplementary Information [file 41467_2025_61797_MOESM1_ESM.pdf]

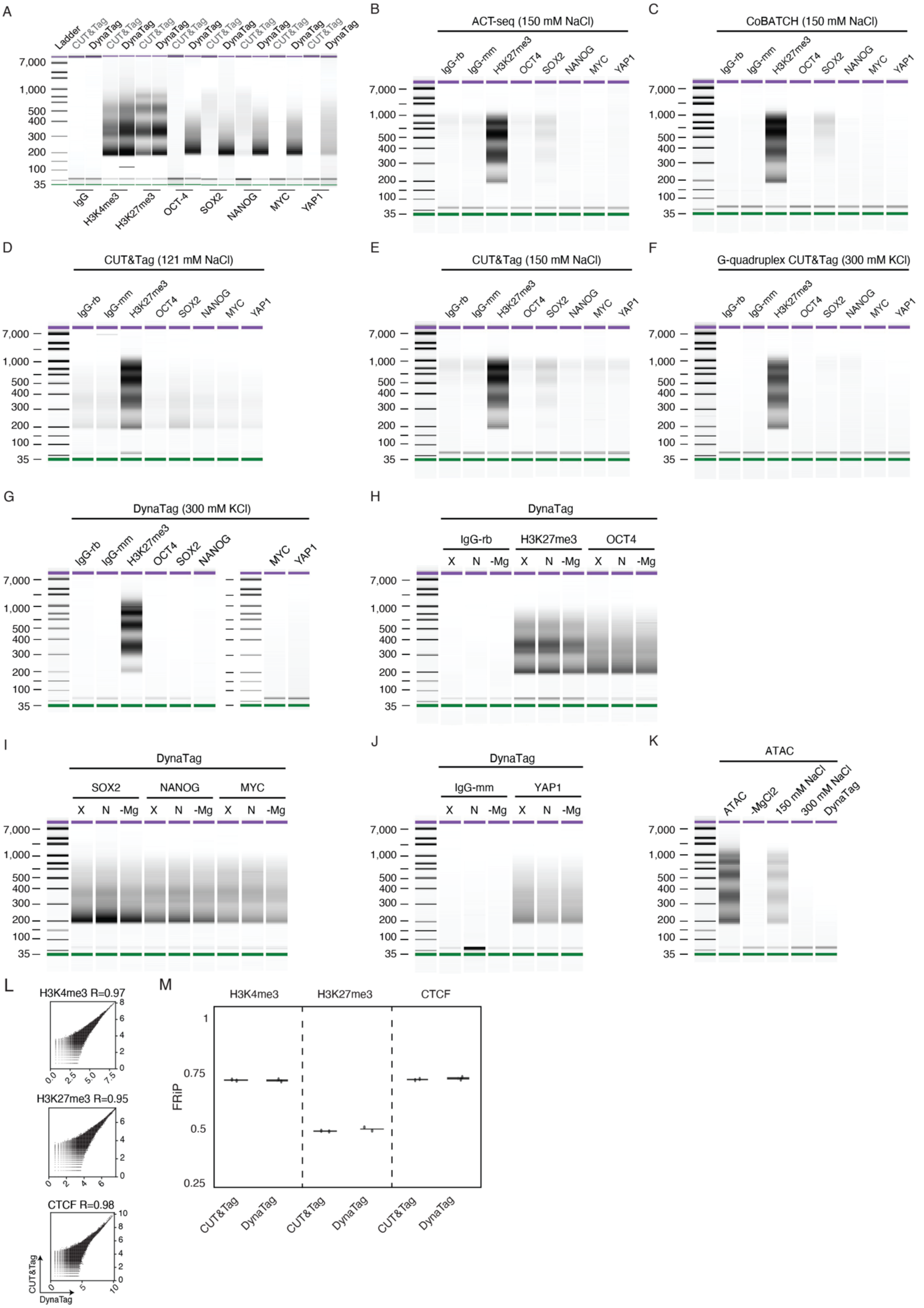

Supplementary Fig. 1: Quality assessment and specificity of the DynaTag physiological buffer. **a**, Bioanalyzer traces for sequencing libraries generated via DynaTag and CUT&Tag for histone modifications and TFs in ESC. **b**, **c**, DynaTag was performed using different nuclei wash procedures of ACT-seq (**b**) and CoBATCH (**c**). **d,** CUT&Tag with 121 mM NaCl wash

buffer. **e,** CUT&Tag with 150 mM NaCl. **f,** DynaTag was performed using the nuclei wash buffer of G-quadruplex CUT&Tag (300 mM KCl). **g,** DynaTag using 300 mM instead of 110 mM KCl in the wash buffer; **h-j,** DynaTag was performed with fixed (X) and native (non-fixed) nuclei (N), and fixed nuclei without magnesium in the DynaTag wash buffer (-Mg) for two IgG control antibodies (rabbit and mouse), H3K27me3, OCT-4, SOX2, NANOG, MYC and YAP1. **k,** Omni-ATAC-seq library preparation using an excess of pA-Tn5 and the tagmentation buffer from the Omni-ATAC-seq protocol (ATAC), the same buffer without $MgCl_2$ (-$MgCl_2$), or with 150 mM and 300 mM NaCl for the CUT&Tag wash buffer, or with the DynaTag buffer. **l,** Pearson correlation of read coverage between DynaTag and CUT&Tag. **m,** FRiP scores of DynaTag vs. CUT&Tag.

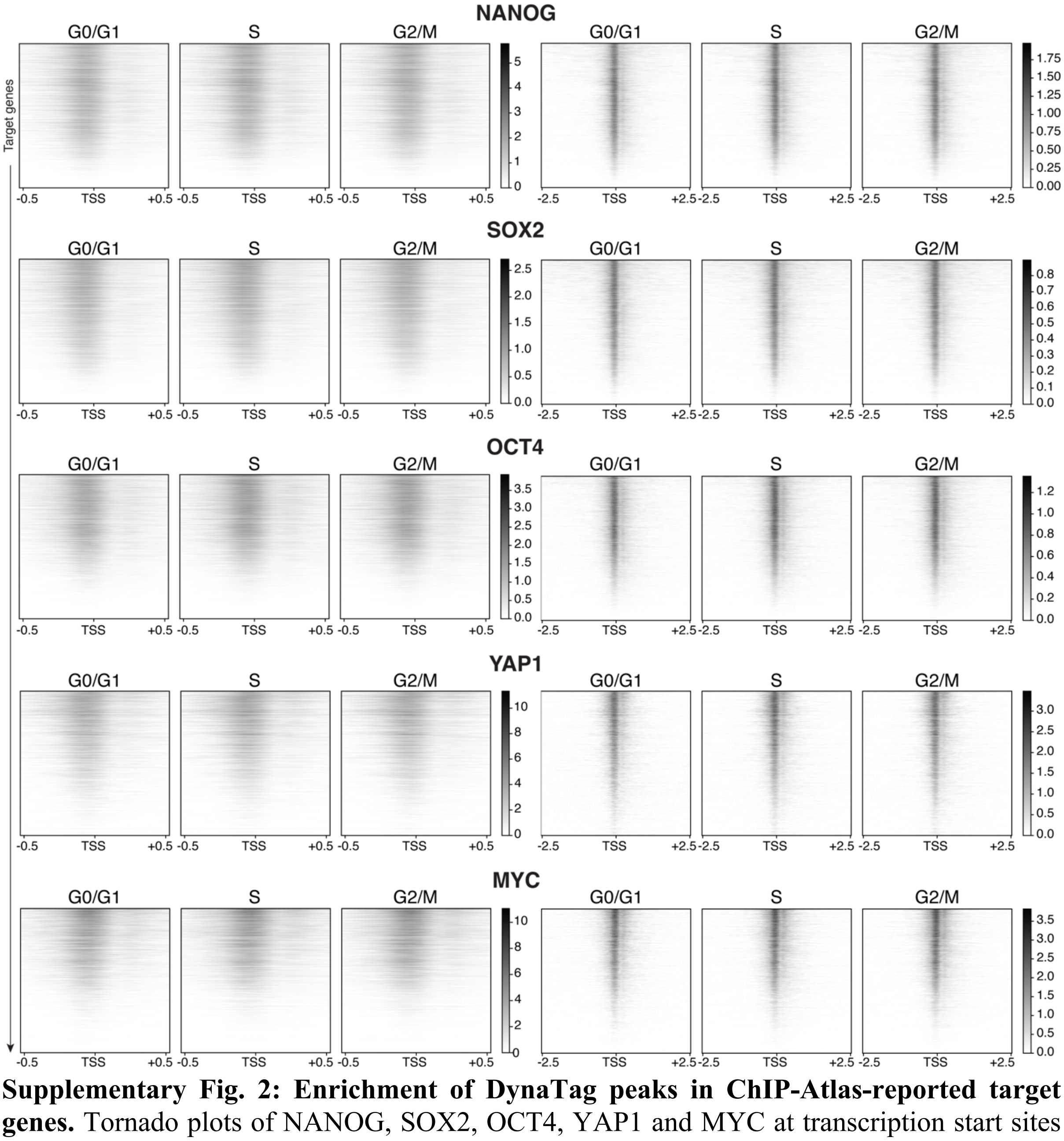

Supplementary Fig. 2: Enrichment of DynaTag peaks in ChIP-Atlas-reported target genes. Tornado plots of NANOG, SOX2, OCT4, YAP1 and MYC at transcription start sites (TSS) of consensus target genes. DynaTag read coverage of transcription factors, 500 bp (left) and 2500 bp (right) upstream and downstream of TSS. Target genes were derived from ChiP-Atlas[22].

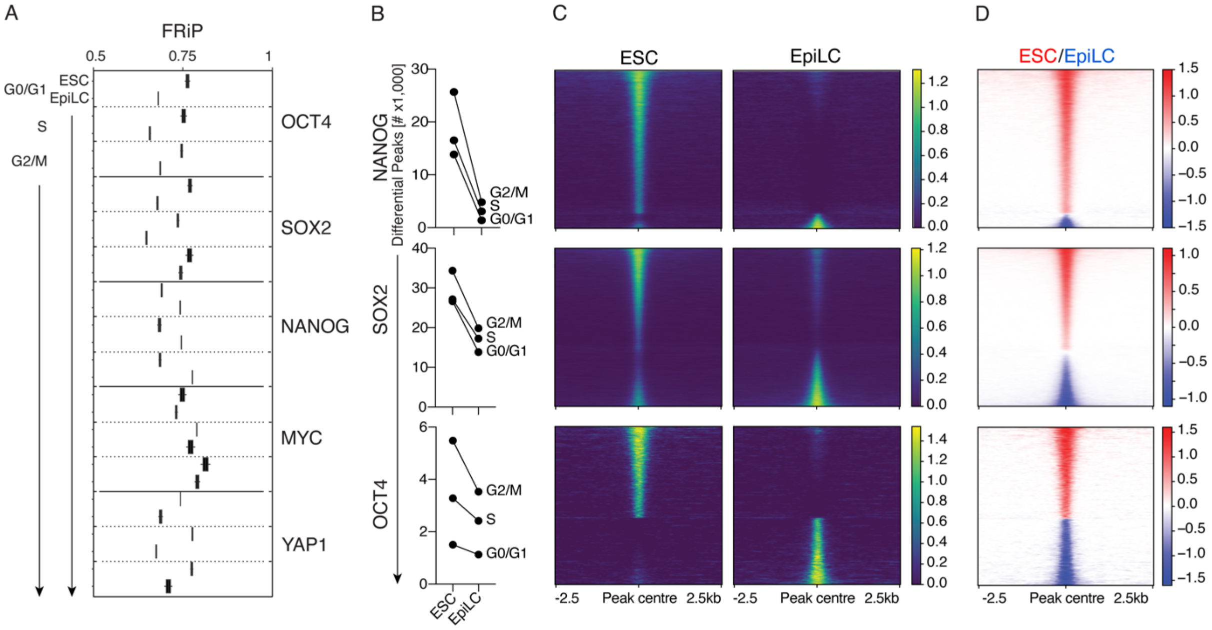

Supplementary Fig. 3: TF dynamics during differentiation of ESCs to EpiLCs. **a,** FRiP score of TFs in ESC and EpiLC per cell cycle phase (G0/G1, S, G2/M) called from DynaTag data. **b**, Differential occupancy analysis (DOA) highlights the number of differentially occupied regions (edgeR) for NANOG, SOX2 and OCT4 in ESC and EpiLC per cell cycle phase using ChIP-seq peaks derived from ChIP-Atlas **c,** Tornado plots of ESC and EpiLC TF bulk DynaTag occupancy showing normalised (cpm) coverage (viridis) and ratios (log2, ESC vs EpiLC) of TF DynaTag at differentially bound (edgeR, >0.5 FC, FDR<0.05) known TF ChIP-seq binding sites. **d,** Summary of the number of differentially occupied regions for each TF. Red indicates increased, blue decreased occupancy of transcription factors in ESC vs EpiLC (ratio in cpm, log2).

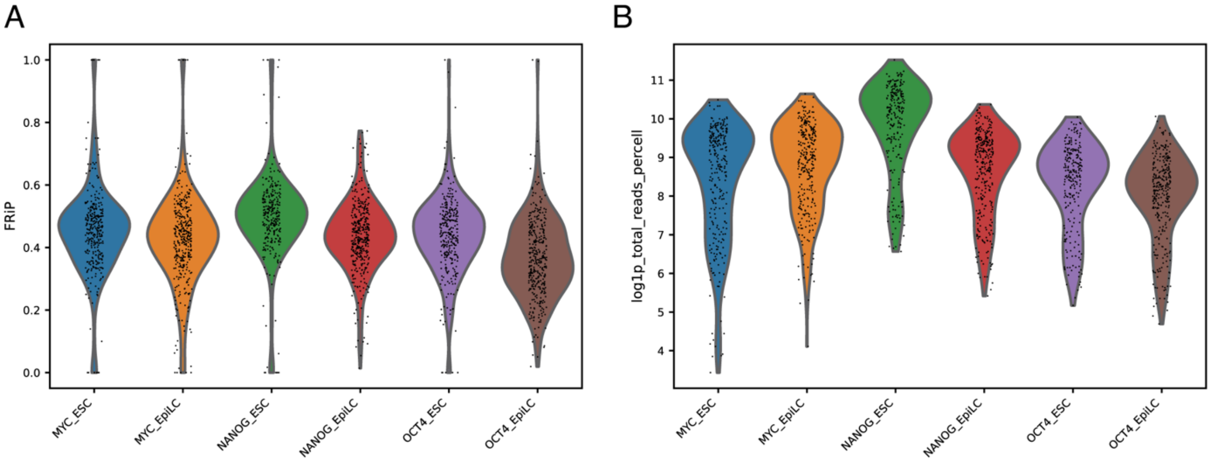

Supplementary Fig. 4: Quality assessment and validation of single-nuclei DynaTag. a, FRiP score distributions of OCT4, NANOG and MYC snDynaTag data in ESC and EpiLC states. **b**, Distribution of log1p, or log(x+1), values for the total number of reads in all peaks across all cells, linked to each TF in ESCs and EpiLCs.

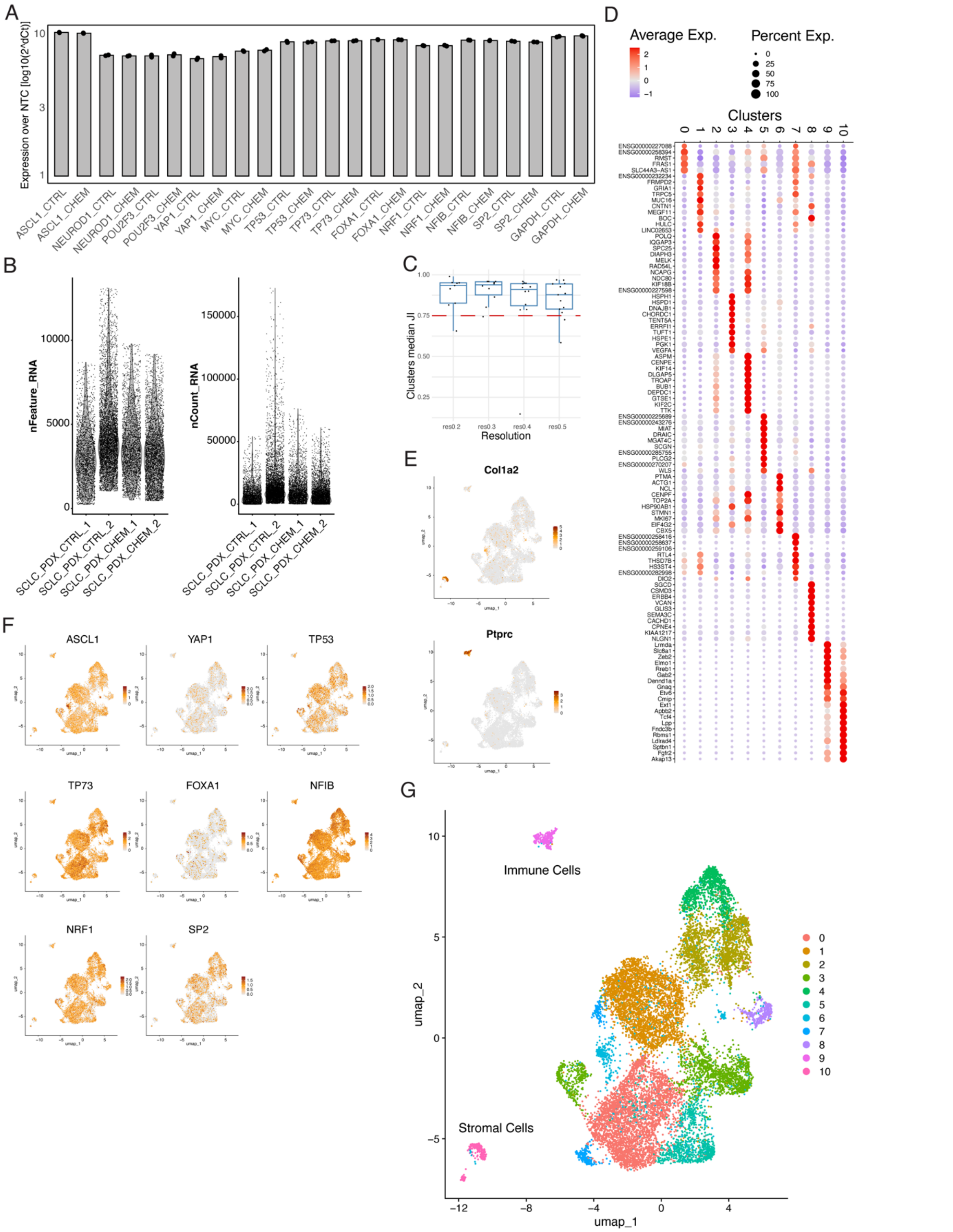

Supplementary Fig. 5: Transcription factor transcripts are detectable in SCLC PDX tumours. a, Bar plot showing the $\log_{10}$-transformed relative RNA expression of the 11 mapped TFs over the respective no-template controls via RT-qPCR in control or chemotherapy sample. GAPDH served as positive control. Data shown from two technical replicates. **b**, Violin plots showing detected genes (features) and counts from scRNA-seq data. **c**, Single-cell cluster stability using Jaccard index. **d,** Dotplot of gene markers defining the 11 clusters. **e,** UMAP with CD45 (*Ptprc*) and *Col1a2* as immune cell and fibroblast marker genes, respectively. **f**, UMAP with expression of the 11 DynaTag-mapped TFs. Missing TFs were not detected. **g,**

UMAP with labelled cell types based on marker gene expression. Scale bars in **e** and **f** indicates normalised expression.

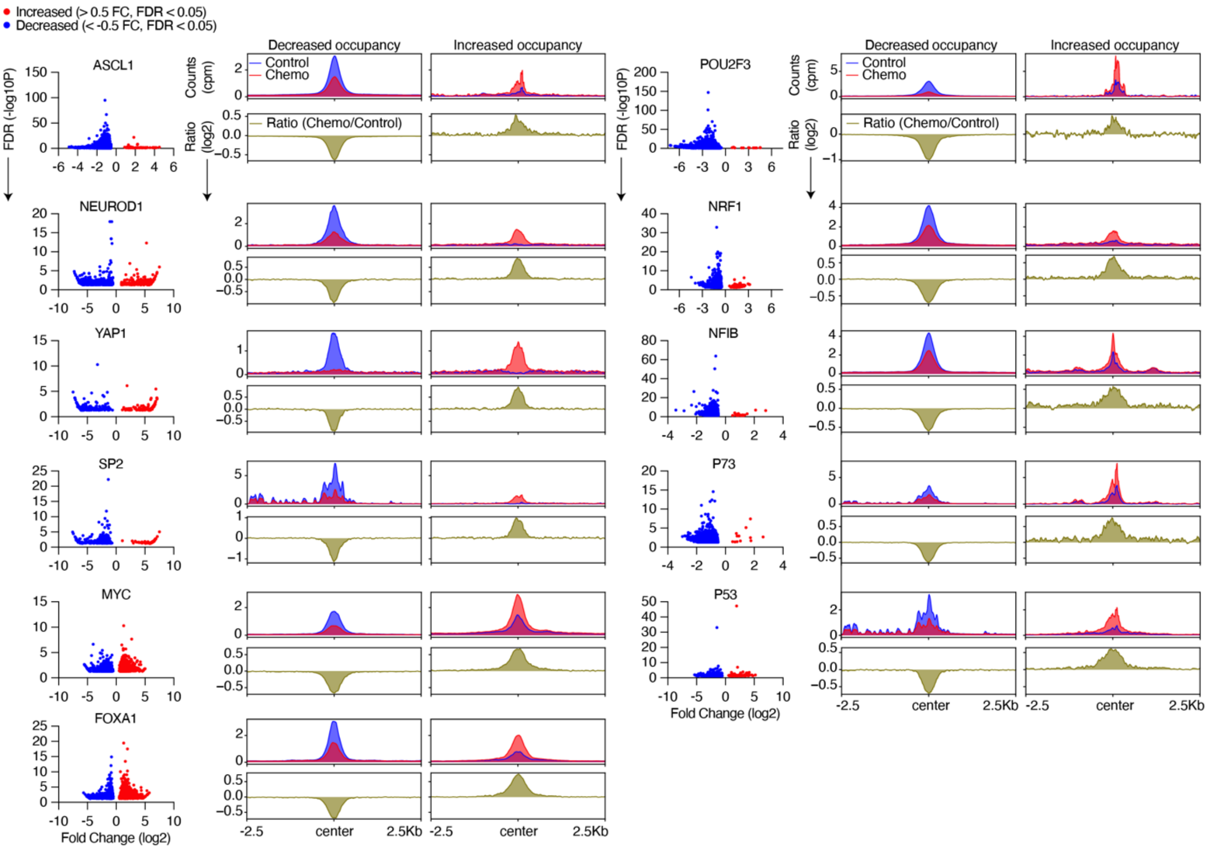

Supplementary Fig. 6: Differentially occupied regions of TFs derived from SCLC PDX DynaTag. For each TF, differentially bound regions (Volcano plot, FDR: <0.05, Fold change: >0.5 or <-0.5, log2), profiles of normalised read coverages (cpm) and ratios of normalised read coverages (log2). Red dots indicate increased, blue dots decreased occupancy in response to chemotherapy. Coverage profiles display increased (red) and decreased (blue) occupancies, as well as ratios (yellow) of chemotherapy treated vs. control treated normalised coverages per TF.

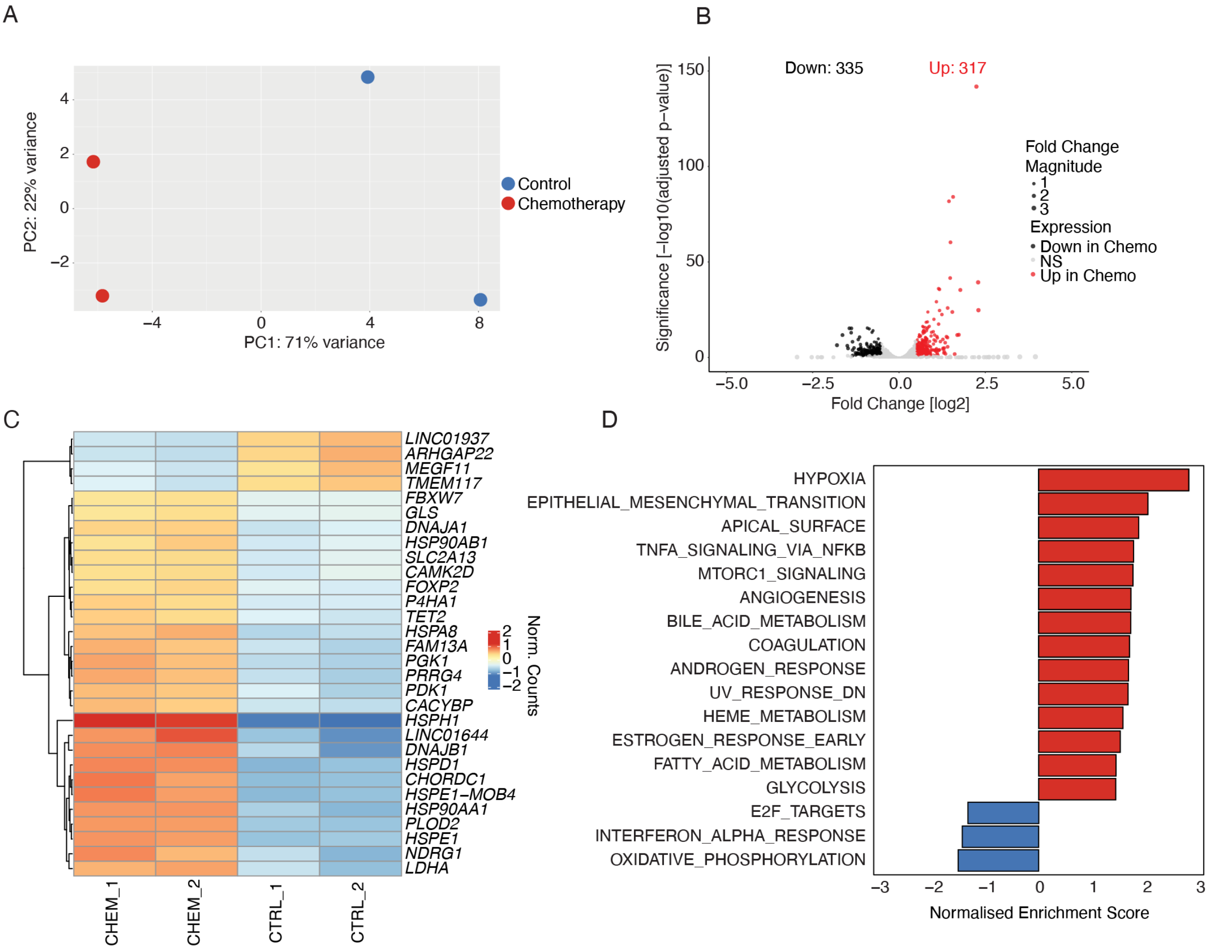

Supplementary Fig. 7: Differential gene expression analysis and GSEA. **a**, PCA of pseudo-bulked scRNA-seq data per replicate. **b**, Volcano plot derived from DESeq2 analysis with up-regulated genes in red and down-regulated genes in black. **c**, Heatmap of top 30 most significant genes. Colour scale in Variance Stabilising Transformation (VST)-normalised data directly derived from DESeq2. **d**, Bar plot for normalised enrichment scores (NES) of significant (NOM p-value <= 0.1) pathways from GSEA. Upon chemotherapy enriched pathways in red; depleted pathways in blue.
