# Supplementary material for: DynaTag for efficient mapping of transcription factors in low-input samples and at single-cell resolution
Source: Nat Commun. 2025 Jul 28;16:6585. doi: 10.1038/s41467-025-61797-9 (PMC12304361; doi:10.1038/s41467-025-61797-9)
Supplement: Supplementary file 2 — Description of Additional Supplementary Information [file 41467_2025_61797_MOESM2_ESM.docx]

Description of Additional Supplementary Files

File name: Supplementary Data 1.xlsx. Description: An overview of chemical reagents used to conduct study.
