# Supplementary material for: DynaTag for efficient mapping of transcription factors in low-input samples and at single-cell resolution
Source: Nat Commun. 2025 Jul 28;16:6585. doi: 10.1038/s41467-025-61797-9 (PMC12304361; doi:10.1038/s41467-025-61797-9)
Supplement: Supplementary file 4 — Reporting Summary [file 41467_2025_61797_MOESM4_ESM.pdf]

nature portfolio

Corresponding author(s): Robert Hänsel-Hertsch

Last updated by author(s): May 30, 2025

Reporting Summary

Nature Portfolio wishes to improve the reproducibility of the work that we publish. This form provides structure for consistency and transparency in reporting. For further information on Nature Portfolio policies, see our Editorial Policies and the Editorial Policy Checklist.

Statistics

For all statistical analyses, confirm that the following items are present in the figure legend, table legend, main text, or Methods section.

n/a	Confirmed	
[ ]	[x]	The exact sample size (n) for each experimental group/condition, given as a discrete number and unit of measurement
[ ]	[x]	A statement on whether measurements were taken from distinct samples or whether the same sample was measured repeatedly
[ ]	[x]	The statistical test(s) used AND whether they are one- or two-sided<br>*Only common tests should be described solely by name; describe more complex techniques in the Methods section.*
[x]	[ ]	A description of all covariates tested
[x]	[ ]	A description of any assumptions or corrections, such as tests of normality and adjustment for multiple comparisons
[ ]	[x]	A full description of the statistical parameters including central tendency (e.g. means) or other basic estimates (e.g. regression coefficient) AND variation (e.g. standard deviation) or associated estimates of uncertainty (e.g. confidence intervals)
[ ]	[x]	For null hypothesis testing, the test statistic (e.g. F, t, r) with confidence intervals, effect sizes, degrees of freedom and P value noted<br>*Give P values as exact values whenever suitable.*
[x]	[ ]	For Bayesian analysis, information on the choice of priors and Markov chain Monte Carlo settings
[x]	[ ]	For hierarchical and complex designs, identification of the appropriate level for tests and full reporting of outcomes
[x]	[ ]	Estimates of effect sizes (e.g. Cohen's d, Pearson's r), indicating how they were calculated

Our web collection on statistics for biologists contains articles on many of the points above.

Software and code

Policy information about availability of computer code

Data collection	Illumina NextSeq 1k, Bio-Rad ChemiDoc MP Imaging System, Bio-Rad CFX Opus Real-Time PCR
Data analysis	bowtie2 (v2.4.1), deeptools (v3.5.1), MACS2 (v2.2.7.1), samtools (v1.13), GenomicRanges (v1.54.1), ggplot2 (v3.5.0), edgeR (v 4.0. 16), DiffBind (Galaxy Version 2.10.0), Seurat (v 5.0.1), Split Pipe (v1.0.6), DESeq2 (v1.42.0), GSEA software (v4.3.3), sctransform (v0.4.1), bedtools (v3.5.1), HOMER2 (v5.1, 7-16-2024), cellranger (v7.0.1), Scanpy (v1.10.4), Seurat (v 5.2.1)

For manuscripts utilizing custom algorithms or software that are central to the research but not yet described in published literature, software must be made available to editors and reviewers. We strongly encourage code deposition in a community repository (e.g. GitHub). See the Nature Portfolio guidelines for submitting code & software for further information.

Data

Policy information about availability of data

All manuscripts must include a data availability statement. This statement should provide the following information, where applicable:

- Accession codes, unique identifiers, or web links for publicly available datasets
- A description of any restrictions on data availability
- For clinical datasets or third party data, please ensure that the statement adheres to our policy

The murine DynaTag, CUT&Tag and RNA-seq data are available at NCBI GEO under accession code GSE273872 [https://www.ncbi.nlm.nih.gov/geo/query/acc.cgi?acc=GSE273872]. The DynaTag and snRNA-seq data related to the PDX model S02730 are available at the European Genome-Phenome Archive, under restricted access by the Data Access Committee University of Cologne, using the accession code EGAS50000001074 [https://ega-archive.org/studies/EGAS50000001074]. The

data generated in this study, underlying the figures, are provided in the Source Data file.

The following published ChIP-seq and CUT&RUN datasets were used for OCT4, SOX2 and NANOG:
SRR10992265 [https://www.ncbi.nlm.nih.gov/sra/?term=SRR10992265]
SRR10992267 [https://www.ncbi.nlm.nih.gov/sra/?term=SRR10992267]
SRR10992266 [https://www.ncbi.nlm.nih.gov/sra/?term=SRR10992266]
SRR23310250 [https://www.ncbi.nlm.nih.gov/sra/?term= SRR23310250]
SRR23310248 [https://www.ncbi.nlm.nih.gov/sra/?term= SRR23310248]
SRR23310227 [https://www.ncbi.nlm.nih.gov/sra/?term= SRR23310227]

The following published ChIP-seq datasets were used for P53:
SRR12884191 [https://www.ncbi.nlm.nih.gov/sra/?term=SRR12884191]
SRR12884190 [https://www.ncbi.nlm.nih.gov/sra/?term=SRR12884190]
SRR27596890 [https://www.ncbi.nlm.nih.gov/sra/?term= SRR27596890]
SRR27596891 [https://www.ncbi.nlm.nih.gov/sra/?term= SRR27596891]

Research involving human participants, their data, or biological material

Policy information about studies with human participants or human data. See also policy information about sex, gender (identity/presentation), and sexual orientation and race, ethnicity and racism.

Reporting on sex and gender	The patient model analyzed in this study was previously described in a cohort (George et al., Nature 2024), which consists of predominantly male patients (66%). This patient model refers to a female patient (ID: S02730)
Reporting on race, ethnicity, or other socially relevant groupings	There was no selection of patients based on race, ethnicity, or other socially relevant groupings
Population characteristics	This current study refers to a model from one patient S02730 (George et al., Nature 2024): gender: female; age at the time of first diagnosis: 64 years.
Recruitment	Patients were diagnosed with Small Cell Lung Cancer. No other specific criteria were applied to recruit patients (no clinical study).
Ethics oversight	University of Cologne

Note that full information on the approval of the study protocol must also be provided in the manuscript.

Field-specific reporting

Please select the one below that is the best fit for your research. If you are not sure, read the appropriate sections before making your selection.

☒ Life sciences ☐ Behavioural & social sciences ☐ Ecological, evolutionary & environmental sciences

For a reference copy of the document with all sections, see nature.com/documents/nr-reporting-summary-flat.pdf

Life sciences study design

All studies must disclose on these points even when the disclosure is negative.

Sample size	2-4
Data exclusions	None
Replication	2-4
Randomization	None
Blinding	None

Reporting for specific materials, systems and methods

We require information from authors about some types of materials, experimental systems and methods used in many studies. Here, indicate whether each material, system or method listed is relevant to your study. If you are not sure if a list item applies to your research, read the appropriate section before selecting a response.

Materials & experimental systems

n/a	Involved in the study
☐	☒ Antibodies
☐	☒ Eukaryotic cell lines
☐	☐ Palaeontology and archaeology
☐	☒ Animals and other organisms
☐	☐ Clinical data
☐	☐ Dual use research of concern
☐	☐ Plants

Methods

n/a	Involved in the study
☒	☐ ChIP-seq
☒	☐ Flow cytometry
☒	☐ MRI-based neuroimaging

Antibodies

Antibodies used	H3K4me3 (abcam, ab8580), H3K27me3 (CST, 9733), CTCF (Merck, 07-729-25UL), OCT4 (acbam, ab181557), SOX2 (CST, 23064), NANOG (CST, 8822), MYC (abcam, ab32072), YAP1 (Santa Cruz, sc-101199), ASCL1 (abcam, ab74065), NEUROD1 (CST, 4373), POU2F3 (Santa Cruz, sc-293402), YAP1 (Santa Cruz, sc-101199), MYC (abcam, ab32072), P53 (abcam, ab1101), P73 (abcam, ab40658), FOXA1 (abcam, ab23738), NRF1 (CST, 46743), NFIB (Invitrogen, PA5-28299), SP2 (abcam, ab229468)
Validation	Validation performed by vendor

Eukaryotic cell lines

Policy information about cell lines and Sex and Gender in Research

Cell line source(s)	Murine Embryonic Stem cells E14
Authentication	Microscopy, Morphology
Mycoplasma contamination	Negative
Commonly misidentified lines (See ICLAC register)	No

Palaeontology and Archaeology

Specimen provenance	*Provide provenance information for specimens and describe permits that were obtained for the work (including the name of the issuing authority, the date of issue, and any identifying information). Permits should encompass collection and, where applicable, export.*
Specimen deposition	*Indicate where the specimens have been deposited to permit free access by other researchers.*
Dating methods	*If new dates are provided, describe how they were obtained (e.g. collection, storage, sample pretreatment and measurement), where they were obtained (i.e. lab name), the calibration program and the protocol for quality assurance OR state that no new dates are provided.*

☐ Tick this box to confirm that the raw and calibrated dates are available in the paper or in Supplementary Information.

Ethics oversight	*Identify the organization(s) that approved or provided guidance on the study protocol, OR state that no ethical approval or guidance was required and explain why not.*

Note that full information on the approval of the study protocol must also be provided in the manuscript.

Animals and other research organisms

Policy information about studies involving animals; ARRIVE guidelines recommended for reporting animal research, and Sex and Gender in Research

Laboratory animals	NOD scid gamma (NSG™) mice
Wild animals	None
Reporting on sex	Female
Field-collected samples	None
Ethics oversight	LANUV, AZ: 84-02.04.2018.A002

Note that full information on the approval of the study protocol must also be provided in the manuscript.

Clinical data

Policy information about clinical studies

All manuscripts should comply with the ICMJE guidelines for publication of clinical research and a completed CONSORT checklist must be included with all submissions.

Clinical trial registration	*Provide the trial registration number from ClinicalTrials.gov or an equivalent agency.*
Study protocol	*Note where the full trial protocol can be accessed OR if not available, explain why.*
Data collection	*Describe the settings and locales of data collection, noting the time periods of recruitment and data collection.*
Outcomes	*Describe how you pre-defined primary and secondary outcome measures and how you assessed these measures.*

Dual use research of concern

Policy information about dual use research of concern

Hazards

Could the accidental, deliberate or reckless misuse of agents or technologies generated in the work, or the application of information presented in the manuscript, pose a threat to:

No	Yes	
☒	☐	Public health
☒	☐	National security
☒	☐	Crops and/or livestock
☒	☐	Ecosystems
☒	☐	Any other significant area

Experiments of concern

Does the work involve any of these experiments of concern:

No	Yes	
☒	☐	Demonstrate how to render a vaccine ineffective
☒	☐	Confer resistance to therapeutically useful antibiotics or antiviral agents
☒	☐	Enhance the virulence of a pathogen or render a nonpathogen virulent
☒	☐	Increase transmissibility of a pathogen
☒	☐	Alter the host range of a pathogen
☒	☐	Enable evasion of diagnostic/detection modalities
☒	☐	Enable the weaponization of a biological agent or toxin
☒	☐	Any other potentially harmful combination of experiments and agents

Plants

Seed stocks	*Report on the source of all seed stocks or other plant material used. If applicable, state the seed stock centre and catalogue number. If plant specimens were collected from the field, describe the collection location, date and sampling procedures.*
Novel plant genotypes	*Describe the methods by which all novel plant genotypes were produced. This includes those generated by transgenic approaches, gene editing, chemical/radiation-based mutagenesis and hybridization. For transgenic lines, describe the transformation method, the number of independent lines analyzed and the generation upon which experiments were performed. For gene-edited lines, describe the editor used, the endogenous sequence targeted for editing, the targeting guide RNA sequence (if applicable) and how the editor was applied.*
Authentication	*Describe any authentication procedures for each seed stock used or novel genotype generated. Describe any experiments used to assess the effect of a mutation and, where applicable, how potential secondary effects (e.g. second site T-DNA insertions, mosiacism, off-target gene editing) were examined.*
